# Randomised clinical trial: the efficacy and safety of pancreatin enteric-coated minimicrospheres (Creon 40000 MMS) in patients with pancreatic exocrine insufficiency due to chronic pancreatitis - a double-blind, placebo-controlled study

**DOI:** 10.1111/j.1365-2036.2012.05202.x

**Published:** 2012-07-04

**Authors:** V Thorat, N Reddy, S Bhatia, A Bapaye, J S Rajkumar, D D Kini, M M Kalla, H Ramesh

**Affiliations:** * Department of Medical Gastroenterology, Poona Hospital & Research CentrePune, India; † Department of Medical Gastroenterology, Asian Institute of GastroenterologyHyderabad ,India; ‡ Department of Gastroenterology Seth G. S. Medical College and KEM HospitalMumbai, India; § Department of Gastroenterology Deenanath Mangeshkar Hospital & Research CentrePune, India; ¶ Division of Gastroenterology Lifeline Multispeciality HospitalChennai, India; ** Manipal AcuNova MHB Clinical Research Centre, Manipal HospitalBangalore, India; †† Department of Gastroenterology,S. R. Kalla Memorial HospitalJaipur, India; ‡‡ Department of Surgical Gastroenterology and Liver Transplantation Lakeshore Hospital & Research CentreKochiIndia

## Abstract

**Background:**

Pancreatic exocrine insufficiency (PEI) results in maldigestion, leading to abdominal pain, steatorrhoea, malnutrition and weight loss.

**Aim:**

To assess the efficacy and safety of pancreatin (Creon 40000 MMS) in treating PEI due to chronic pancreatitis (CP).

**Methods:**

This was a 1-week, double-blind, randomised, placebo-controlled, parallel-group, multicentre study in India. Men and women ≥18 years of age with proven CP and PEI [defined as a coefficient of fat absorption (CFA) ≤80% during run-in phase] were randomised 1:1 to pancreatin or placebo (two capsules orally per main meal, one with snacks). The primary outcome measure was change in CFA from baseline to end of double-blind treatment (analysis of covariance).

**Results:**

Of 62 patients randomised (34 pancreatin, 28 placebo), 61 completed treatment; one patient in the placebo arm withdrew consent before completion. Patient characteristics were similar in both groups except for the proportion of men (pancreatin 82% vs. placebo 68%). Patients receiving pancreatin had a statistically significant greater improvement in fat absorption from baseline to the end of double-blind treatment compared with those receiving placebo, with a least squares mean change (95% CI) in CFA of 18.5% (15.8–21.2) vs. 4.1% (1.0–7.2), respectively. This resulted in a treatment difference of 14.4% (10.3–18.5); *P* = 0.001. Patients receiving pancreatin also had a statistically significant greater improvement in nitrogen absorption and greater reductions in mean stool fat, stool frequency and stool weight compared with those receiving placebo. Treatment-emergent adverse events occurred in 12 patients on pancreatin and in seven on placebo; none led to study discontinuation.

**Conclusions:**

The results provide evidence for the efficacy of pancreatin (Creon 40000 MMS) in patients with pancreatic exocrine insufficiency due to chronic pancreatitis, and confirm that this formulation is well tolerated, with a good safety profile, at the dose administered.

## Introduction

Chronic pancreatitis (CP) is an inflammatory disorder of the pancreas that causes progressive, irreversible pancreatic injury. The pathophysiological characteristics include fibrosis of pancreatic tissue, pancreatic duct dilation, calcifications in the pancreatic ducts or parenchyma and endocrine and exocrine dysfunction.^1-3^ CP results from complex interactions between multiple genetic and environmental factors.^1-3^ The TIGAR-O classification system categorises risk factors that may predispose an individual to CP: Toxic-metabolic (including alcohol and smoking); Idiopathic (no clear risk factor); Genetic; Autoimmune; Recurrent and severe acute pancreatitis; Obstructive.^1^ In industrialised regions, alcohol use has long been considered the dominant risk factor for CP,^1^ although in a recent study in the United States, 56% of cases were classified as nonalcohol related or idiopathic.^4^ In the Asia-Pacific region, most cases of CP are idiopathic.^3, 5, 6^ Tropical calcific pancreatitis (TCP) is an idiopathic form of CP that occurs mainly in developing countries in tropical regions^3,^^7,^and appears to be very common in southern India.^6, 7^ There are no clear diagnostic criteria for TCP,^6^ but it is characterised by absence of alcohol use, recurrent abdominal pain in childhood, earlier age of onset (usually 10–30 years), insulin-dependent diabetes in most cases (termed fibrocalculous pancreatic diabetes) and a high frequency of large intraductal calcifications.^7, 9^

The primary symptoms of CP are abdominal pain, which may be accompanied by nausea and vomiting, and the clinical signs associated with pancreatic exocrine insufficiency (PEI); later complications include diabetes and pancreatic cancer.^1, 2, 3^ PEI is defined as inadequate delivery of pancreatic enzymes into the small intestine, causing maldigestion of food. The most common symptoms of PEI-associated maldigestion are abdominal pain, steatorrhoea, malnutrition and weight loss.^10^ PEI appears to occur later in the disease course in early-onset idiopathic CP compared with alcoholic CP,^1994^ and may occur even later in Indian patients with idiopathic CP,^12^ which may result in differences in the frequency and severity of PEI in Asian-Pacific populations compared with Western regions. In addition, the lower fat content of the diet in these patients relative to Western populations may also result in a lower frequency of clinical symptoms associated with fat maldigestion.^7^

Regardless of its aetiology, the clinical standard for the management of PEI is pancreatic enzyme replacement therapy (PERT). Pancreatin (pancrelipase) enteric-coated minimicrospheres (Creon MMS; Abbott, Hannover, Germany) is a well-studied PERT that is available worldwide in various formulations and dosage forms that differ in terms of strength in lipase units. Pancreatin has been shown to be effective for the treatment of PEI due to CP or pancreatic surgery, with a good tolerability profile, in two double-blind, randomised, placebo-controlled trials.^13,^^14^ In a small observational study enrolling patients with TCP, pancreatin treatment for 6 months significantly improved flatulence, abdominal pain, diarrhoea and steatorrhoea.^15^ Pancreatin has also been shown to be effective in treating PEI due to cystic fibrosis (CF) in randomised controlled trials and open-label studies.^16-22^

This double-blind, randomised, placebo-controlled trial was performed in India to assess the efficacy and safety of the pancreatin (Creon) 40000 MMS formulation in patients with PEI due to CP. The primary objective was to demonstrate superior efficacy of pancreatin over placebo using the primary outcome measure of change in coefficient of fat absorption (CFA) from baseline to the end of the 1-week, double-blind phase. Secondary endpoints included nitrogen absorption, stool characteristics, clinical symptoms and safety.

## Materials and Methods

### Study design

This was a 1-week, double-blind, randomised, placebo-controlled, parallel-group, multicentre study to assess the efficacy and safety of pancreatin 40000 MMS (ClinicalTrials.gov number NCT00705978) that was conducted at 11 centres in India; nine centres contributed patients. The study was conducted in compliance with EU Clinical Trial Directive 2001/20/EC, the International Conference on Harmonization guideline for Good Clinical Practice, and the ethical principles of the Declaration of Helsinki, and was approved by an independent ethics committee that complied with local regulatory requirements. Voluntary written consent was obtained from all subjects before screening.

The study comprised a prestudy screening (Visit 1), followed by a 2-week run-in period, a 1-week, double-blind, randomised, placebo-controlled phase, then a 51-week open-label extension (Figure). This paper reports data from the double-blind period only; outcomes of the open-label extension will be reported separately. During Week 1 of the run-in period, subjects were hospitalised for 5 days during which 96-h nutritional recording and 72-h stool collection were carried out to determine baseline CFA and coefficient of nitrogen (CNA) values. During this time, subjects did not receive PERT. Nutrition recording started 24 h before stool collection, which began at 0:00 hour on the second day of nutrition recording and ended at 24:00 hours on the last day of nutrition recording. Patients were advised by a dietician how to ingest at least 100 g dietary fat/day during this period. At the end of stool collection and nutritional recording (Visit 2), subjects were allowed to take their normal dose of PERT until randomisation. On Day 1 (Visit 3), which occurred within 1 week of Visit 2, subjects with a baseline CFA ≤ 80% were randomised to the double-blind phase. Nutrition recording and stool collection were conducted on Days 4–7 in the same way as in the run-in period. Day 8 (Visit 4) marked the end of the double-blind phase.

**Figure 1:**
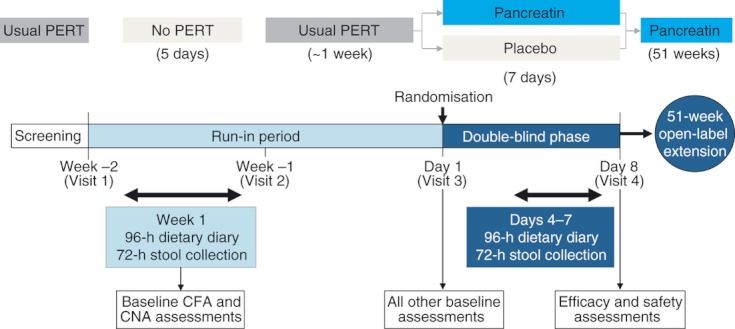
Study design. PERT, pancreatic enzyme replacement therapy; CFA, coefficient of fat absorption; CNA, coefficient of nitrogen absorption.

### Study participants

The diagnosis of CP was made using endoscopic retrograde cholangiopancreatography, endosonography, ultrasonography indicative of calcifications or duct dilatation, other suitable imaging techniques (e.g. plain film radiography, computed tomography) and/or histology. Patients were required to have PEI as determined by a CFA ≤ 80% during the run-in phase. Other inclusion criteria were ≥18 years of age and, if women, nonlactating and not of childbearing potential or having agreed to practice an approved contraception method throughout the study, which had been used for at least 3 months prior to screening.

Exclusion criteria included: medical conditions that could interfere with the study or study drug; endocrine disease other than diabetes; major surgery except gall bladder removal or appendectomy; ileus or acute abdomen; any type of malignancy involving the digestive tract in the past 5 years; investigational drugs within 30 days prior to study entry; current excessive intake of alcohol or drug abuse; and hypersensitivity to porcine proteins or pancreatin.

Concomitant use of other PERT preparations was prohibited. Medications influencing duodenal pH [H_2_-receptor antagonists, antacids, sucralfate, proton pump inhibitors (PPIs), prostaglandins, anticholinergic agents, or somatostatin], drugs acting on gastric emptying and drugs interfering with bile secretion could be given at a stable dose throughout the study. Other medications that did not interfere with the study or study drug were allowed if deemed necessary.

### Treatment

Patients were enrolled by the participating centres and randomised 1:1 to pancreatin or placebo according to the randomisation list generated by the Pharmaceutical Supplies department of Abbott, using a block size of 8. Study medication was supplied with the randomisation codes printed on the labels.

Patients received pancreatin (Creon) 40000 MMS (Abbott) or placebo capsules orally. The dose was two capsules with each main meal (3 meals per day) and one capsule with snacks (2–3 snacks per day) for a total of six to nine capsules per day. The first dose was to be taken with the first meal on Day 1. Creon 40000 MMS is provided as a hard, gastro-resistant capsule containing gastro-resistant pellets (Minimicrospheres) that provide 400 mg pancreatin (pancreatic enzyme powder) per capsule, corresponding to 40 000 Ph. Eur. units lipase. Thus, the dose in lipase units was 80 000 Ph. Eur. units per main meal and 40 000 Ph. Eur. units per snack. The actual lipase value at release was approximately 45 000 units/capsule.

Medication was dispensed in aluminium-aluminium blister packs with 10 capsules per blister. Blinding was maintained by the identical appearance of study medication and placebo.

### Efficacy assessments

The primary efficacy outcome measure was the change in CFA from baseline to the end of double-blind treatment. CFA was determined by the equation CFA = 100*[(mean fat intake − mean stool fat)/mean fat intake]. Stool samples were analysed at the Department of Biochemistry and Biophysics, St John's Medical College, Bangalore, India) and stool fat was determined according to the van de Kamer method.^23^ Missing values for CFA at baseline or end of the double-blind period were not imputed.

Secondary efficacy endpoints were change from baseline to end of the double-blind phase in CNA, stool characteristics, clinical symptoms, clinical global impression (CGI) of disease symptoms, body weight and body mass index (BMI). CNA was determined by the equation CNA = 100*[(mean nitrogen intake − mean stool nitrogen)/mean nitrogen intake]. Mean stool weight, stool fat and stool nitrogen (g/day) were determined from the net weight/fat/nitrogen in the 72-h stool sample. Mean fat and nitrogen intake were determined from the 96-h dietary diaries by a dietician using suitable software; nitrogen intake was determined by calculating the mean protein intake recorded then multiplying by 0.16 (the average nitrogen content of a polypeptide chain). Clinical symptoms were assessed by investigators by asking subjects to provide information on number of stools per day, stool consistency (hard, formed/normal, soft, watery), flatulence (none, mild, moderate, or severe) and abdominal pain (none, mild, moderate, or severe). The CGI of disease symptoms was determined by investigators by asking patients to rate symptoms at each visit as none (absent), mild (present, but not bothersome), moderate (bothersome), severe (interfered with normal activity), or incapacitating (prevented normal activity).

### Safety evaluation

Adverse events (AEs) were recorded using standard medical terminology and assigned to the terms in the Medical Dictionary for Regulatory Activities (MedDRA) version 12.1 (International Federation of Pharmaceutical Manufacturers and Associations, Geneva, Switzerland). AEs were considered treatment-emergent (TEAEs) if they started or worsened with or after the first study drug administration and on the last day plus a gap period of 1 day. An AE was considered drug-related if the investigator judged it possibly or probably related to the experimental drug, or if this assessment was missing.

Physical examination and assessment of vital signs was carried out at baseline and at the end of the double-blind phase. Laboratory safety parameters were measured at baseline and then at Week 26 and are therefore not reported here.

### Statistical analysis

Data were analysed by DATAMAP GmbH, Freiburg, Germany using sas Release 9.1. The sample size was determined from the results of two previous studies on pancreatin in subjects with PEI, one of which has been published.^14^ A difference of 16% [residual standard deviation (s.d.) 18%] between the pancreatin and placebo groups for the full analysis (FA) sample was considered a conservative estimate for the expected difference in CFA analysed using analysis of covariance (ancova). Assuming a normal distribution and a two-sided Type I level of 0.05, 28 patients in each treatment group were needed to achieve 90% statistical power. Therefore, 35 patients per treatment group were planned to account for missing data in 20% of subjects.

The safety sample comprised all subjects allocated to treatment who took at least one dose of pancreatin or placebo. Efficacy analyses were performed on the FA sample, defined as subjects in the safety sample who had at least one post-baseline assessment of any efficacy measurement. For the primary outcome measure, analyses using the per protocol (PP) sample were also carried out, defined as patients in the FA sample with no major protocol deviations.

Efficacy outcomes are described by standard summary statistics. For the primary outcome measure of CFA, treatment groups were compared using ancova with the baseline value as covariate and treatment as factor. Least squares (LS) means and 95% CI were calculated and *P*-values were estimated using the F-test. Similar exploratory analyses were carried out for the CNA and stool fat. *Post hoc* exploratory subanalysis of change in CFA from baseline to end of the double-blind phase was carried out according to concomitant use of PPIs vs. no use of PPIs.

## Results

The study was performed between June 2008 and May 2010. Patient disposition is shown in Figure. Of the 62 patients randomised, 61 completed the double-blind phase; one patient from the placebo group withdrew consent before completion.

**Figure 2:**
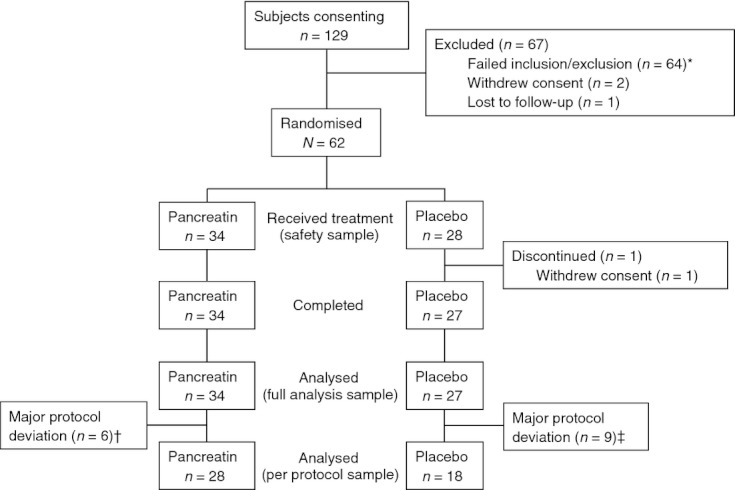
Patient disposition. * All had a coefficient of fat absorption >80%. †Insufficient essential efficacy data (*n* = 2), randomisation envelope lost (*n* = 2), insufficient evidence of PEI (*n* = 2). ‡Insufficient essential efficacy data (*n* = 4), randomisation envelope lost (*n* = 3), insufficient evidence of PEI (*n* = 2), insufficient exposure to study medication during double-blind period (*n* = 2), no post-baseline efficacy data (*n* = 1), use of prohibited prior/concomitant medication (*n* = 1). Some patients had more than one protocol deviation.

Patient characteristics were similar across both groups (Table) although the proportion of men was higher in the pancreatin group (82.4%) than in the placebo group (67.9%). Two subjects in each group were randomised to treatment even though their baseline CFA was >80%.

**Table 1:** Subject characteristics at baseline (safety sample)

Parameter	Pancreatin (*n* = 34)	Placebo (*n* = 28)
Age (years)		
Mean ± s.d.	42.6 ± 11.1	43.2 ± 10.4
Median (min/max)	44 (19/62)	44 (18/60)
Gender, *n* (%)		
Male	28 (82.4)	19 (67.9)
Female	6 (17.6)	9 (32.1)
Race, *n* (%)		
Asian	34 (100)	28 (100)
Weight (kg), mean ± s.d.	51.1 ± 9.9	49.0 ± 7.5
BMI (kg/m^2^), mean ± s.d.	19.1 ± 3.1	18.4 ± 2.5
Method used to prove CP, *n* (%)[*]		
Ultrasonography	22 (64.7)	15 (53.6)
Endosonography	4 (11.8)	2 (7.1)
ERCP	5 (14.7)	5 (17.9)
Other	16 (47.1)	18 (64.3)
Time since last proof of CP (years), mean ± s.d.	1.8 ± 3.4	2.0 ± 1.8
Comorbid condition, *n* (%)	31 (91.2)	22 (78.6)
Diabetes mellitus	18 (52.9)	12 (42.9)
Previous pancreatic surgery (any)	8 (23.5)	10 (35.7)
Abdominal pain	5 (14.7)	3 (10.7)
Hypertension	5 (14.7)	3 (10.7)
Steatorrhoea	4 (11.8)	2 (7.1)

BMI, body mass index; CP, chronic pancreatitis; ERCP, endoscopic retrograde cholangiopancreatography; s.d., standard deviation.

* In some patients more than one test was used to diagnose CP, therefore the values do not add up to 100%. Most tests in the ‘other’ category were computed tomography scans.

During the double-blind period, 26 (76.5%) patients in the pancreatin group and 23 (82.1%) in the placebo group took concomitant medications. The most common were drugs for peptic ulcer or gastro-oesophageal reflux disease [12 (35.3%) in the pancreatin group and 10 (35.7%) in the placebo group]; insulin or insulin analogues [12 (35.3%) and 6 (21.4%), respectively]; blood glucose-lowering drugs other than insulin [9 (26.5%) and 6 (21.4%), respectively] and calcium [4 (11.8%) and 5 (17.9%), respectively].

The mean duration of double-blind treatment was 7.8 days (median 8 days) in both groups. The range was 7–9 days in the pancreatin group and 5–10 days in the placebo group.

### Efficacy analysis

Patients randomised to pancreatin had a significantly greater improvement in CFA from baseline to end of double-blind treatment compared with placebo (Table 2), with an LS mean change from baseline of 18.5% vs. 4.1%, respectively (14.4% treatment difference; *P* = 0.001). A similar outcome was observed in CFA change from baseline to end of the double-blind period using the PP sample, with a mean treatment difference of 13.7% (95% CI, 9.1–18.2; *P* = 0.001).

**Table 2:** Least squares mean and 95% CIs for change from baseline to end of double-blind phase (ancova with baseline as covariate and treatment as a factor; full analysis sample)

	Pancreatin (*n* = 34)*	Placebo (*n* = 27)*	Treatment difference	*P*-value
CFA (%)	18.5 (15.8, 21.2)	4.1 (1.0, 7.2)	14.4 (10.3, 18.5)	0.001
CNA (%)	4.7 (3.0, 6.5)	0.8 (–1.3, 2.9)	4.0 (1.2, 6.7)†	0.005
Stool fat (g/day)	−19.8 (−23.0, −16.6)	−4.3 (−8.0, −0.6)	−15.5 (−20.4, −10.6)	0.001

ancova, analysis of covariance; CFA, coefficient of fat absorption; CNA, coefficient of nitrogen absorption.

* Owing to missing stool samples, two subjects in the pancreatin group and three subjects in the placebo group were excluded from the CFA and stool fat analysis; four subjects in the pancreatin group and seven subjects in the placebo group were excluded from the CNA analysis.

† Slight difference in value compared with pancreatin–placebo due to rounding.

Significantly greater improvements in the CNA and stool fat were also observed in the pancreatin group vs. the placebo group using exploratory ancova analysis (Table 2). Unadjusted mean values for the CFA, CNA and stool characteristics are summarised in Table 3. Mean fat intake did not change significantly in either group over the double-blind period: 110.3 g/day at baseline and 115.9 g/day at the end of the double-blind period in the pancreatin group, and 108.7 g/day and 108.2 g/day, respectively, in the placebo group. Similarly, mean nitrogen intake did not change appreciably during the double-blind period: 10.8 g/day at baseline and 11.6 g/day at end of the double-blind period in the pancreatin group, and 11.4 g/day and 11.5 g/day, respectively, in the placebo group.

**Table 3:** Unadjusted mean ± standard deviation values at baseline and end of double-blind period for CFA, CNA, and stool characteristics (full analysis sample)*

	Pancreatin (*n* = 34)*			Placebo (*n* = 27)*				
*n*		*n*	
CFA (%)				
Baseline	34	66.5 ± 14.1	27	67.0 ± 14.0
End of double- blind phase	32	86.1 ± 7.5	24	72.9 ± 11.5
CNA (%)				
Baseline	32	78.8 ± 10.0	23	79.7 ± 7.2
End of double- blind phase	32	83.8 ± 6.9	24	81.7 ± 7.3
Stool fat (g/day)				
Baseline	34	37.3 ± 17.0	27	35.3 ± 14.9
End of double- blind phase	32	16.2 ± 9.6	24	30.0 ± 13.8
Stool nitrogen (g/day)				
Baseline	32	2.0 ± 0.5	23	2.0 ± 0.4
End of double- blind phase	32	1.7 ± 0.3	24	1.9 ± 0.4
Stool weight (g/day)				
Baseline	34	714 ± 284	27	678 ± 200
End of double- blind phase	31	423 ± 208	24	565 ± 212
Stool frequency per day				
Baseline	34	2.9 ± 1.7	27	2.6 ± 0.9
End of double- blind phase	34	2.6 ± 2.4	27	2.5 ± 1.2

CFA, coefficient of fat absorption; CNA, coefficient of nitrogen absorption.

*Owing to missing stool samples at baseline and/or end of double-blind treatment, some patients in the full analysis sample are missing from these analyses.

**Figure 3:**
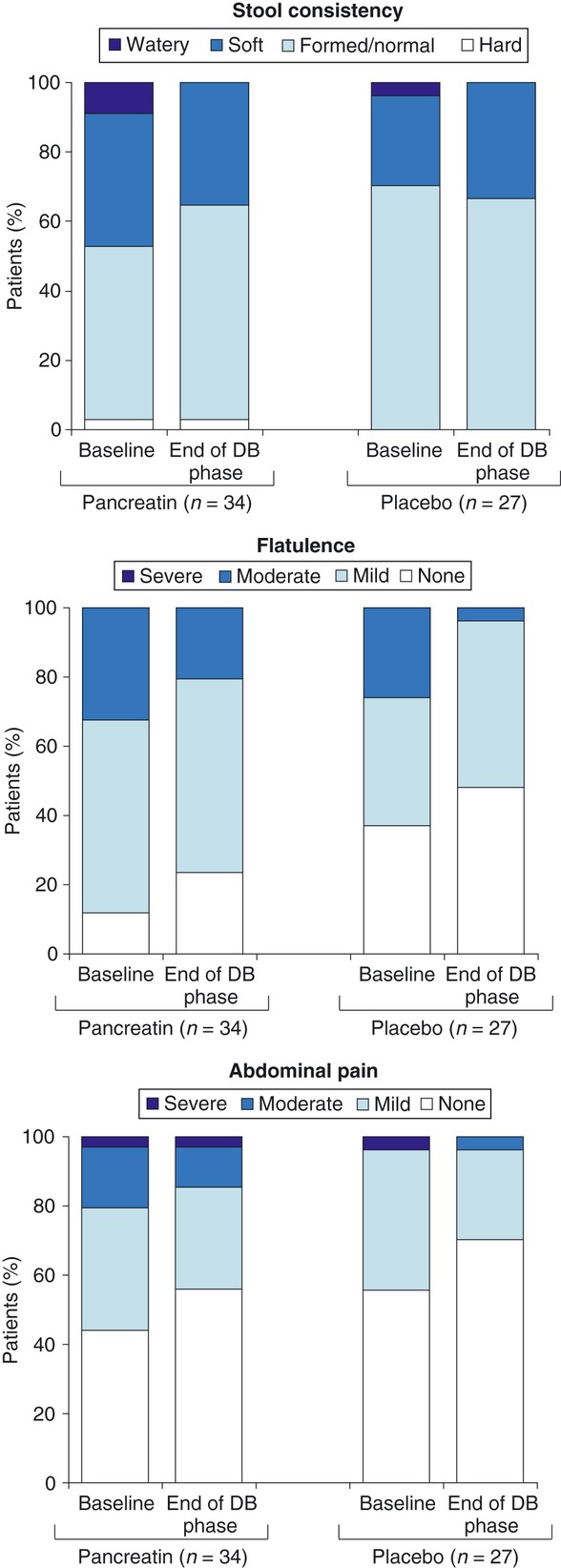
Clinical symptoms (full analysis sample). DB, double-blind.

**Figure 4:**
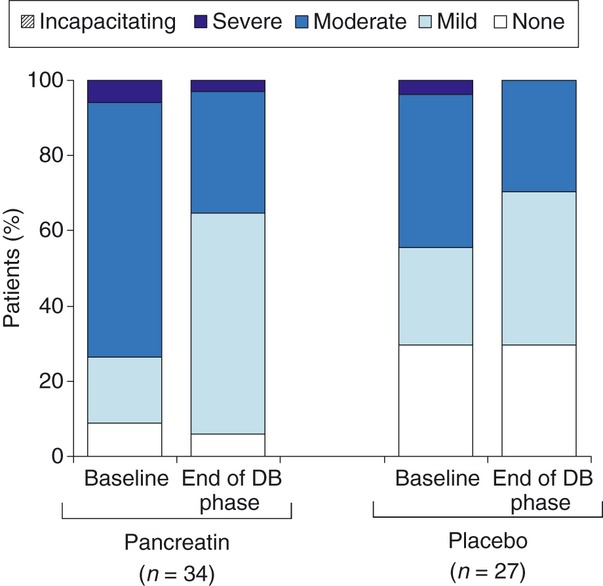
Clinical global impression of disease symptoms (full analysis sample). No patients reported incapacitating symptoms. DB, double-blind.

Changes in clinical symptoms are shown in Figure 3. Clinical symptoms appeared to be less severe in the placebo group at baseline. Stool consistency improved slightly in the pancreatin group and remained about the same in the placebo group. Flatulence and abdominal pain improved slightly in both groups. No relevant differences in any of the clinical symptoms were observed between treatment groups at the end of the double-blind period. Greater improvements were observed in the pancreatin group vs. the placebo group for the Clinical Global Impression of disease symptoms (Figure 4). At baseline, the proportion of patients with moderate/severe symptoms was higher in the pancreatin group vs. the placebo group.

Changes in body weight (mean ± s.d. change 0.2 ± 1.7 kg and 0.1 ± 1.0 kg, respectively) and BMI (0.1 ± 0.6 kg/m^2^ and 0.0 ± 0.4 kg/m^2^, respectively) were similar in the pancreatin vs. the placebo groups.

An exploratory subanalysis indicated that in patients taking pancreatin, mean ± s.d. CFA values were similar at the end of the double-blind period in those taking concomitant PPIs (86.2 ± 7.3%) and in those not taking PPIs (86.0 ± 7.8%). The mean change in CFA was lower in those taking PPIs than in those not taking PPIs (15.9 ± 5.3% vs. 21.2 ± 13.1%); however, the mean baseline CFA values were higher in subjects who used PPIs (70.3 ± 8.7% vs. 64.5 ± 16.1%). Statistical comparisons were not carried out for these sub-analyses.

### Safety

Treatment-emergent adverse events (TEAEs) occurred in 35.3% patients in the pancreatin group and 25.0% in the placebo group. The most common are summarised in Table 4; as expected in this patient population, gastrointestinal disorders were the most frequent TEAEs. One patient in the pancreatin group experienced a TEAE that was considered probably related to treatment by the investigator (abdominal discomfort).

**Table:** Treatment-emergent adverse events occurring in at least two patients in either treatment group by preferred term (safety sample)

	Pancreatin (*n* = 34)	Placebo (*n* = 28)
Patients with one or more TEAE, *n* (%)	12 (35.3)	7 (25.0)
TEAEs with possible relationship to study drug, *n* (%)	1 (2.9)	0 (0)
Gastrointestinal disorders, *n* (%)	9 (26.5)	1 (3.6)
Abdominal pain	3 (8.8)	0 (0.0)
Hyperchlorhydria	2 (5.9)	0 (0.0)
Abdominal discomfort	2 (5.9)	0 (0.0)
Vomiting	2 (5.9)	0 (0.0)
Musculoskeletal and connective tissue disorders, *n* (%)	2 (5.9)	2 (7.1)
Arthralgia	2 (5.9)	0 (0.0)

TEAE, treatment-emergent adverse event.

During the double-blind period, there were no TEAEs leading to study termination, no treatment-emergent serious adverse events (SAEs) and no severe TEAEs. One subject in the pancreatin group experienced an SAE, 1 day before the start of the double-blind period (gastritis), and one subject in the placebo group experienced an SAE, 4 days before the start of the double-blind period (lower respiratory tract infection). No deaths occurred during the study.

No clinically relevant changes were observed in vital signs during the double-blind period.

### Discussion

This was the first randomised, controlled trial assessing the pancreatin (Creon) 40000 MMS formulation in patients with PEI due to CP. In this 1-week, randomised, double-blind, placebo-controlled, parallel-group study, patients receiving pancreatin had a statistically significant greater improvement in fat absorption from baseline to the end of double-blind treatment compared with placebo, as measured using the CFA. Patients receiving pancreatin also had a significantly greater improvement in nitrogen absorption (CNA) compared with placebo, and greater reductions in mean stool fat, stool frequency and stool weight. Baseline CFA and CNA values were similar in both groups, and there were no between-group differences in fat and nitrogen intake at baseline or at the end of the double-blind period.

There were no relevant differences between groups regarding change in clinical symptoms (stool consistency, flatulence and abdominal pain) during the double-blind period. As clinical symptoms were not present or mild in most patients at baseline in both groups, it is unlikely that large changes would be seen over 1 week.

Greater improvements in the CGI of disease symptoms were observed in the pancreatin vs. the placebo group, although CGI ratings were slightly better in the placebo group at baseline. No changes were seen in body weight or BMI. These parameters would not be expected to change significantly over the course of 1 week; long-term changes will be analysed and described for the open-label period separately.

A subanalysis according to PPI use indicated that CFA values at the end of the double-blind period were similar in patients taking concomitant PPIs and in those not taking concomitant PPIs, suggesting that PPI use does not affect the efficacy of pancreatin. The greater increase from baseline in CFA in patients not taking PPIs can be explained by the lower baseline CFA in this subgroup.

Pancreatin was well tolerated with a good safety profile, and no unexpected AEs were observed. The overall incidence of TEAEs was higher in the pancreatin group; however, only one patient in the pancreatin group had a TEAE that was considered possibly related to treatment by the investigator. Gastrointestinal disorders were the most common TEAEs observed and were more frequent in the medical history of subjects in the pancreatin group at baseline (30% vs. 25% in the placebo group).

The outcomes of this study compare favourably with studies of other pancreatin formulations in patients with PEI due to CP. A double-blind, randomised, placebo-controlled, parallel-group trial conducted in the United States and Eastern Europe investigated the efficacy and safety of pancreatin (Creon 12 000) in 54 patients with PEI due to CP or pancreatic surgery.^13^ That study had a similar design to the present study, but patients received placebo during a 5-day run-in period prior to the 7-day double-blind phase. Mean CFA and CNA values on pancreatin at the end of the double-blind period (86% and 79%, respectively) were similar to those reported in this study, and the mean changes from baseline were significantly greater with pancreatin vs. placebo (CFA: 32% vs. 9%, *P* < 0.0001; CNA: 35% vs. 9%, *P* = 0.0005). The greater magnitude of change from baseline is likely due to lower baseline CFA and CNA values in the pancreatin group (54% and 46%, respectively), which indicates more severe PEI at baseline compared with this study. Different CP aetiology (e.g. a higher proportion of idiopathic CP or TCP) could be a contributing factor to the higher baseline CFA and CNA values observed in this study, indicating less severe PEI. Differences in the dietary content of the two patient populations may also have contributed to these baseline differences. Although patients were advised by a dietician in both studies to ensure ingestion of at least 100 g fat/day, the dietary fat intake in the Whitcomb *et al*. study was approximately one-third higher than that of the patients in this study and the protein intake approximately double, which may lead to greater malabsorption in patients not receiving PERT. Similar improvements in stool frequency were seen in both studies. In the Whitcomb *et al*. study, statistically significant improvements in flatulence and stool consistency were observed with pancreatin vs. placebo. As might be expected in patients with more severe PEI, clinical symptoms appeared to be more severe at baseline compared with this study. In the 6-month, open-label extension of the Whitcomb *et al*. study, the CFA and CNA were not assessed, but from baseline to end of open-label treatment, there was a significant improvement in stool frequency (1.0 ± 1.3 stools/day; *P* < 0.001) and body weight (2.73 ± 3.35 kg; *P* < 0.0001).^24^ Improvements in clinical symptoms (abdominal pain, stool consistency and flatulence) and CGI of disease symptoms were also observed.

In a separate, double-blind, placebo-controlled trial in the United States, 27 patients with PEI due to CP were randomised to pancreatin (Creon 10) or placebo for 2 weeks following a 2-week placebo run-in phase.^14^ CFA values at the end of the double-blind phase were 87% on pancreatin and 68% on placebo, similar to the present study. The changes from baseline (37% and 12%; *P* = 0.0185) were again of larger magnitude than in this study owing to lower baseline CFA values (50% pancreatin and 56% placebo). Significant reductions in stool frequency were observed and physician-assessed GCI scores were significantly improved with pancreatin vs. placebo.

The on-treatment CFA and CNA values in this study were also comparable to those seen in a double-blind, randomised, cross-over study of another available PERT formulation [Zenpep (pancrelipase) Delayed-Release Capsules].^25^ In that study, the on-treatment CFA was 88.9% with low-dose PERT and 89.9% with high-dose PERT (both *P* < 0.001 vs. placebo run-in value of 81.7%), and the CNA was 84.1% and 85.4%, respectively (both *P* < 0.001 vs. 78.1% placebo).^25^ However, unlike our study, patients were not required to meet a predefined level of PEI severity at baseline.

The dosing schedule used in this study of 80 000 Ph. Eur. lipase units per main meal and 40 000 per snack is similar to that used in the Whitcomb *et al*. study (72 000 USP lipase units per main meal and 36 000 per snack). This is an established recommended dose for the treatment of PEI due to CP; in clinical practice dose should be tailored to the individual patient's needs. These doses are within dosing ranges recommended by other authors.^26, 27^

The patient population in this study [mean age 43 years (range 18–62)] was younger than those assessed in the other two studies described above [51 (32–75) and 51 (31–74), respectively].^13, 14^ This may be due to differing aetiology of CP in this Indian patient population compared with the United States and European studies, with a greater frequency of idiopathic CP and TCP in this study. Detailed information on the aetiology of CP for the patients enrolled in this study is not available. The proportion of patients having undergone pancreatic surgery was slightly lower in the pancreatin group (24%) than in the placebo group (36%). However, we do not consider this to be a limitation, as the focus of this study was the treatment of PEI rather than CP or pancreatic surgery. All patients enrolled in this study had the presence of PEI confirmed based on a CFA ≤ 80%; the aetiology of the underlying disease is not expected to affect the efficacy or safety of pancreatin in treating PEI. Furthermore, the previous study by Whitcomb *et al*. found no difference in pancreatin efficacy when comparing the CFA outcome in patients with CP vs. patients who had undergone pancreatic surgery.2010

The findings of this study are applicable to all other currently available pancreatin MMS formulations as they differ in strength in terms of lipase. This study adds to the existing clinical evidence supporting the efficacy and safety of pancreatin and provides additional information on its efficacy and safety in a patient population from a different geographical region to those enrolled in previous clinical studies.

Taken together, these findings provide strong evidence for the efficacy of pancreatin (Creon) 40000 MMS in patients with PEI due to CP, and confirm that this formulation is well tolerated at the dose administered.
